# How can cervical screening meet the needs of vulnerable women? A qualitative comparative study with stakeholder perspectives from seven European countries

**DOI:** 10.1136/bmjopen-2024-090631

**Published:** 2025-01-25

**Authors:** Rikke Buus Bøje, Marc Bardou, Keitly Mensah, Raquel Rico Berrocal, Laura Bonvicini, Giusy Iorio, Noemi Auzzi, Diana Taut, Nicoleta-Monica Pașca, Anna Tisler, Kerli Reintamm, Anneli Uusküla, Margarida Teixeira, João Firmino-Machado, Mariana Amorim, Ines Baia, Nuno Lunet, Yulia Panayotova, Tatyana Kotzeva, Irina Todorova, Berit Andersen, Pia Kirkegaard, CBIG-SCREEN Consortium, Firmino Machado

**Affiliations:** 1 University Research Clinic for Cancer Screening, Randers Regional Hospital, Randers, Denmark; 2 CIC-P INSERM 1432, Centre Hospitalier Universitaire de Dijon, Dijon, France; 3 Early Detection, Prevention & Infections Branch, International Agency for Research on Cancer/World Health Organization, Lyon, France; 4 Centre d’études des mouvements sociaux, EHESS, BAGNOLET, France; 5 Servizio Epidemiologia, Azienda USL-IRCCS di Reggio Emilia, Reggio Emilia, Italy; 6 Qualitative Research and Citizen Science Unit, Azienda USL-IRCCS di Reggio Emilia, Reggio Emilia, Italy; 7 Instituto per lo Studio, la Prevenzione e la Rete Oncologica, Osservatorio Nazionale Screening, Florence, Italy; 8 Psychology, Universitatea Babes-Bolyai Facultatea de Psihologie si Stiinte ale Educatiei, Cluj-Napoca, Romania; 9 Institute of Family Medicine and Public Health, University of Tartu, Tartu, Tartumaa, Estonia; 10 EPIUnit, Instituto de Saúde Pública da Universidade do Porto, Porto, Portugal; 11 Laboratório para a Investigação Integrativa e Translacional em Saúde Populacional, Universidade do Porto, Porto, Porto, Portugal; 12 Health Psychology Research Center, Sofia, Bulgaria; 13 Deparment of Clinical Medicine, Aarhus Universitet Faculty of Health, Aarhus, Denmark

**Keywords:** Mass Screening, Community-Based Participatory Research, PUBLIC HEALTH, Primary Prevention, QUALITATIVE RESEARCH, Gynaecological oncology

## Abstract

**Abstract:**

**Objective:**

This study explored and compared stakeholder perspectives on enhancements to cervical cancer screening for vulnerable women across seven European countries.

**Design:**

In a series of Collaborative User Boards, stakeholders were invited to collaborate on identifying facilitators to improve cervical cancer screening.

**Setting:**

This study was part of the CBIG-SCREEN project which is funded by the European Union and targets disparities in cervical cancer screening for vulnerable women (www.cbig-screen.eu). Data collection took place in Bulgaria, Denmark, Estonia, France, Italy, Portugal and Romania.

**Participants:**

Represented stakeholders at various levels, including user representatives (vulnerable women), healthcare professionals, social workers, programme managers and decision makers.

**Methods:**

14 meetings lasting 2 hours each were held in these seven countries between October 2021 and June 2022. The meetings were audio or video recorded, transcribed and translated into English for qualitative framework analysis.

**Results:**

We engaged 120 participants in the Collaborative User Boards. Proposed solutions targeted both provider and system levels. In all countries, fostering trusting relationships between vulnerable women and social or healthcare professionals, coupled with community outreach for awareness and access to testing was a consistent recommendation. Participants in Estonia, Denmark, France, Italy, Portugal and Romania advocated for tailoring healthcare services to meet the unique needs of vulnerable populations through a holistic approach. In Bulgaria and Romania, participants advocated for the need to secure free access, from screening to follow-up, and emphasised the need for organised screening with target population screening registries.

**Conclusion:**

The study offers insights into stakeholders' recommendations for enhancing cervical cancer screening services for vulnerable women across seven European countries. Despite variations in the implementation level of population-based screening programmes, the imperative to optimise outreach and proximity work to improve cervical cancer screening resonated across all countries.

**STRENGTHS AND LIMITATIONS OF THIS STUDY**

- Data were collected in a varied sample of countries representing the Northern, Southern, Eastern and Western parts of Europe.
- Data underwent a framework analysis which is suitable for comparing similarities and differences in large qualitative cross-country datasets.
- The sample size was robust (n=120), thereby increasing transferability of study results.
- A limitation was the lack of comparison of population groups which hampers the transferability of suggested solutions tailored to each population.
- A limitation was the cross-country variation in the number of participants.

## Introduction

 Implementation of comprehensive cervical cancer screening (CCS) programmes and high coverage of Human papillomavirus (HPV) vaccination have brought European and other high-income countries closer to the realisation of the WHO’s goal of eliminating cervical cancer (CC).^1^ However, there are large disparities in women’s attendance in CCS across Europe and globally.

A recognition of the need to take action and secure access to CCS for women from vulnerable groups in Europe paved the way for the CBIG-SCREEN project, which aims to tackle inequity in CCS for vulnerable women (www.cbig-screen.eu) and in which the present study is nested.

An expected outcome of CBIG-SCREEN is CCS interventions tailored to the specific needs of vulnerable women. This article is the second of two based on the same study within CBIG-SCREEN (for the first study, see Bøje *et al* 2024).^2^

In all European countries but predominantly in Southern and Eastern Europe, women from disadvantaged populations, whether for socio-cultural, economic or geographical reasons, are less likely to attend CCS programmes which increases their risk of CC.^3^

Substantial evidence underscores barriers to CCS for vulnerable women in how CCS is organised, provided and related to vulnerable women’s life situations.^24^,^6^ A scoping review, where most of the included studies were conducted in the UK, revealed several barriers. These included financial obstacles, distrust in the healthcare system and limited access to information about CC and CCS procedure, coupled with women’s lack of emphasis on preventive healthcare.^6^ Factors promoting participation are less often described. The review found that increased access, the provision of culturally appropriate information and improving the experience of the screening process are facilitators for attendance in CCS. At the healthcare system level, organised screening programmes, as opposed to opportunistic screening, have been shown to enhance screening participation, whereas the latter has faced criticism for expending community resources without substantial impact.^7^ Furthermore, organised screening appears to promote equity in access compared with opportunistic screening.^8^ This suggests that both proximity work and outreach work are important to increase access and participation. While proximity work primarily focuses on reducing geographical barriers to access, outreach work focuses on actively engaging with individuals and communities to promote healthcare access and empowerment.^9 10^

In Europe, most countries offer population-based CCS, yet variations in programme implementation and other contextual factors result in varying levels of participation. Self-sampling, where women are provided with a self-test to self-collect cervico-vaginal material at home has proven to increase participation.^11^ However, the participation rate depends on the invitation strategy and the existence of pathways for the procession of the sample. Tranberg and colleagues^12^ found that women from all socioeconomic groups, and in particular, immigrants and women from lower socioeconomic groups benefited from self-sampling when receiving a test kit in the mailbox rather than opt-in screening, where they had to order the kit themselves.

Despite the need for specific attention to the challenges faced by vulnerable women in CCS, a pan-European survey showed that only six countries have policies designated to broaden coverage among subgroups of vulnerable populations.^13^

This study involved stakeholders across countries, professions and social boundaries to provide knowledge of their perspectives on the improvement of CCS for vulnerable women in Europe.

The aim of this study was to elicit and compare stakeholders’ suggestions on how to improve CCS for vulnerable women and perspectives on self-sampling in seven European countries.

## Methods

This study has a different aim but uses the same dataset and method as previously reported in Bøje *et al* 2024^2^ where a more detailed description of the method can be found. We used a qualitative approach and established Collaborative User Boards (CUBs). CUBs were designed to explore and document the views and experiences of stakeholders representing different social positions and professions and to promote mutual learning and engagement. Three stakeholder levels were involved: the micro level representing the users and target groups, the meso level representing professionals working with the target groups or with screening programmes and the macro level representing policy and decision makers.

### Setting and recruitment

The study was conducted in Bulgaria, Denmark, Estonia, France, Italy, Portugal and Romania. The seven countries were selected among the partner countries in CBIG-SCREEN and represent the Northern, Southern, Eastern and Western parts of Europe to provide diversity in the data collection. Among the study countries, Denmark has the lowest incidence of CC and mortality rates per 100 000 women (10,2/2,2) and Romania has the highest (22,6/9,57).^14^ The total risk of poverty varies from 17% in Denmark (lowest) to 34,4% in Romania (highest).^15^ Of these countries, only Bulgaria does not provide a publicly funded organised population-based CCS programme or a screening registry. In Bulgaria, Denmark, Estonia and France, health insurance serves as a source of funding in CCS.^16^ Besides variations in screening policies, poverty rates and access to healthcare services countries were selected to represent most of the European region: East (Bulgaria and Romania, North (Denmark and Estonia), West (France and Portugal) and South (Italy).

Eligibility of participants was guided by a survey, also nested in the CBIG-SCREEN, revealing the most vulnerable subgroups in each country as defined by experts and programme managers and based on risk factors such as belonging to groups with low socioeconomic status, living with HIV virus and being female sex workers.^13^

### Data collection and analysis

Between February and June 2022, a series of CUB meetings were held to discuss the barriers and possible solutions in CCS for vulnerable women in all seven countries. The way the CUB meetings were organised and whether the barriers and solutions were discussed separately or in the same meeting varied from country to country. Either way, the discussions of solutions were connected to the participants’ identification of barriers.

The research teams followed an inductive approach adapting to participants’ discussions. However, all research teams were told to ask specifically about perceptions on the use of self-sampling as a solution to improve CCS.

The local research teams translated the audio and video recordings of the CUB meetings into English, and then securely transmitted them to the first author via an encrypted connection. Subsequently, the transcriptions were imported into NVivo, a qualitative data management software, for analysis.^17^ The whole dataset was used for analysis, because possible solutions were discussed throughout the CUB meetings regardless of the headline of the meeting. We conducted the analysis inspired by the framework analysis method by Ritchie and Spencer,^18^ following the steps of initial coding, development of an analytical framework, summarising and abstracting themes and charting these for comparison. Finally, the themes and subthemes were mapped into clusters of countries based on similarities of suggested solutions. A cluster is constituted by similar perceptions of a suggested solution within more than one subtheme between specific countries. For validation and understanding of context, the interpretation of data was discussed throughout the process with coauthors from all local research teams.

### Patient and public involvement

In this study, emphasis was put on the perspectives of stakeholders impacted by the lack of optimal CCS for vulnerable women. Their perspectives have the potential to influence the development of future policies within CCS. At the CUB meetings, the participants were encouraged to share their suggestions on how to improve CCS for vulnerable women. In this way, the participants were involved as participants with lived experience.

## Results

We enrolled a total of 120 participants, including 39 micro-, 63 meso- and 18 macro-level stakeholders (table 1).

**Table 1:** Level of representation and distribution of participants across the seven countries

Level of representation	Occupation/Sub-groups	Participants per country	Gender
Micro (n=39)	Women from ethnic minorities, women with substance use disorder, women with low socio- economic status, sexworkers, homeless women, women with physical disability and including intersectionality	Bulgaria (n=5)Denmark (n=8)Estonia (n=8)France (n=3)Italy (n=1)Portugal (n=4)Romania (n=10)	Female (n=39)
Meso (n=63)	Medical doctors, nurses, midwifes, front office operators employed at hospitals, clinics, in screening services, and in primary care non-governmental organisations and associations for vulnerable populations: managers and social workers, health- or cultural mediators Employees and managers in healthcare insurance and screening services.	Bulgaria (n=12)Denmark (n=9)Estonia (n=7)France (n=7)Italy (n=9)Portugal (n=7)Romania (n=12)	Female (n=43)Male (n=20)
Macro (n=18)	Representation from screening services at regional and national level, healthcare and social services departments at ministerial, regional or municipality level and the media.	Bulgaria (n=1)Denmark (n=4)Estonia (n=6)France (N/A))Italy (n=6) Portugal (n=1) Romania (N/A)	Female (n=14)Male (n=4)

We identified suggestions from the CUBs for the improvement of CCS at the provider and system level (see online supplemental material). Suggestions for improvement at the provider level included education and training of providers to improve cultural sensitivity and communication skills when working with vulnerable women, maintaining technical skills and expanding the workforce to carry out the test and raise awareness about CCS. In addition, suggestions for improvement included an emphasis on outreach and proximity work where healthcare professionals or mediators should be present in the community, supporting women throughout the CCS pathway and relational work. It was suggested that family and community should play an important role to motivate women to attend CCS.

At the system level, it was emphasised to tailor the approaches to vulnerable women, to prioritise prevention, to involve healthcare decision makers, to develop a regulatory framework, to foster cross-sector collaboration and to implement a population-based screening programme. Additionally, free screening for all women including women without health insurance was suggested. Awareness about CCS should be increased by verbal information provided face-to-face, via social media or television and written information provided through leaflets on websites or at posters placed in central locations. Peer-to-peer learning, testimonials, national campaigns, nudging and shared decision-making tools were also suggested to increase awareness. Suggested guidelines for information and invitations included clear, culturally sensitive language, provided in multiple local languages, to reduce stigma and be easily accessible. Suggested content included an overview of the CCS process overview, its free nature, consequences of the disease, treatment efficacy and comprehensive cancer and screening information.

Suggestions to improve accessibility included invitations with a prescheduled appointment and quick response codes with further information and invitations sent via social media messaging channels. Suggestions to enhance access for vulnerable women included deploying mobile units, integrating CCS into existing healthcare and social services, offering drop-in screening and providing screenings at local community facilities. In addition, recommendations focused on addressing practical concerns such as parking, simplified registration and less bureaucratic processes. Other suggestions included allowing women to choose a female doctor for the test, receiving test results via email, post or text message, and ensuring results are presented in easily understandable language. Immediate reactions to the introduction of self-sampling in the CCS were mainly expressed by the representatives of vulnerable women and were both positive and negative. Positive responses ranged from seeing the method as more convenient and less anxiety-provoking, beneficial for women with special needs or a history of trauma, substance abuse or sexual assault, to considering it empowering and potentially life-saving. Negative reactions included fear of misuse, a preference for specialist care, concerns about missing out on other gynaecological observations, exclusion of women with mental health problems and low literacy, mistrust in authority-sent materials and doubts about the test validity.

The aim of this study was to elicit and compare stakeholders’ suggestions on how to improve CCS for vulnerable women in seven European countries. We identified similarities and differences in the participants’ suggestions for improvement in clusters of countries. In all countries (cluster 1), the participants made suggestions for improvement in terms of an increase in outreach and proximity work, training of providers to maintain instrumental skills and skills to provide awareness and easy access to CCS. In cluster 2 countries, which included Denmark, Estonia, France, Italy, Portugal and Romania, the importance of focusing on the needs of vulnerable women and collaboration and integration of services was emphasised. In cluster 3 countries constituted by Bulgaria, Romania, Estonia and France, participants highlighted the need to establish a culture of prevention, implement population-based CCS, legal frameworks and a screening registry and provide free CCS for all women.

### Cluster 1: suggestions for improvement from participants in all countries

Participants emphasised the importance of outreach activities to promote awareness and improve access to CCS. This involved engaging health mediators, social workers, personnel from associations for vulnerable women and various healthcare professionals including general practitioners, pharmacists, midwives, technicians and operators. Professionals providing awareness should be present at shelters, in mobile vans/units, in migrant reception centres, drop-in centres and when attending healthcare services for other purposes. A Portuguese participant suggested that providing access through mobile units, vans and offices within the community could be a solution reach the women (table 2, excerpt 1). Danish participants discussed the possibility of raising awareness among the clients of the substance abuse clinic (table 2, excerpt 2). Bulgarian participants specifically emphasised the role of health mediators in building trust and reducing fear among vulnerable women (table 2, excerpt 3). Health mediators could also organise initiatives promoting community education, peer learning and group sessions for women only, and Danish participants suggested meetings with health professionals present in shelters and emphasised the importance of motivation to participate such as a free meal (table 2, excerpt 4). French participants specifically suggested training nurses as this is a large professional group with a majority of females, thus removing barriers related to the gender of the test person. It was also suggested that other professionals should offer testing during vulnerable women’s visits to healthcare and social services (table 2, excerpt 5). In addition, it was emphasised by Romanian participants to train providers to maintain or enhance their medical and communication skills and engage in awareness initiatives (table 2, excerpt 6).

**Table 2:** Examples of excerpts

Subtheme	Excerpts
Outreaching— proximity work	1. **Portuguese participant (meso**): "I defend proximity work and, obviously, if we are talking about vulnerable populations. We can go even further and have all these solutions in mobile teams and be close with trucks, with offices…"
	2. **Danish participant 1 (macro**): “Once a year, the women attending the substance user clinic are offered a health check-up performed by a nurse. They could ask about screening as well. But they would probably need some education”.**Danish participant 2 (macro): “**It is a way to make aware of it”.**Danish participant 1 (macro**): “It is a possibility. We offer the women a talk once a year where they can talk about their concerns and where we also talk about their sexual health. But it is difficult to ask in the right way”.
Health mediators to provide awareness	3. **Bulgarian participant (meso): “**Well, the extended family can help and the mediator. The role of the mediator is to explain”.
Face-to-face awareness	4. **Danish participant 1 (micro**): “Maybe, someone could do a lecture about screening”**Researcher:** “Yes, but would you attend if for instance we made a lecture at the shelter?”**Danish participant 2 (micro):** “If you for instance serve food at the same time, then I think that more young people would attend. I think it would motivate people to come and enjoy themselves during a lecture”.
Train nurses to perform the test	5. **French participant (meso):** “If you want to erode the gender of the practitioner, you can just change the practitioner, there are probably female nurses that you could train to be able to”.Researcher: “What you are saying is good to build the capacity of nurses in cervical cancer screening to do the smear test. because there are many nurses and most of them are women”.
Continuous training of medical doctors to perform the test	6. **Romanian participant (meso):** “GPs are different, too. They are trained differently, so they will not all do the same things. How shall I say this. In screening, we have trained and educated the population for years, we should also train and educate doctors for years”.
Integration of vulnerable women’s needs	7. **Danish participant (meso):** “I insist that this is a problem created by humans, which can be changed if we decide it. We have a tendency to say that it is impossible to integrate the needs of vulnerable people. Of course we can. We just have to do it and allocate the resources”.
	8. **Italian participant (meso):** “It is important to draw the attention to the services that deal with specific categories of women also to issues for which the service is not in charge, but which are still important for women. Beyond the specificity of the paths and services, the woman must be taken care as a whole and that therefore also the specialized services will have to develop a greater responsibility also towards treatment and prevention paths”.
	9. **French participant (meso): “**Generally speaking, you have shared decision support tools, you have international criteria for the types of information you can have in them and …What is important for me is that it is the patients' values that are reflected and which serves as a basis for a discussion of shared decisions. If we are dealing with a vulnerable population, we should go towards this”.
Collaboration and integration across services	10. **Italian participant (meso):** “Obviously, promoting screening in the most vulnerable segments of the population is much more complicated and in this case the invitation letter or multilingual information brochures are not enough. We need to do extensive work in the reception centres where migrants come, seek the help of cultural mediators who can explain how screening is organized, how to join and convince people that it is good for their health”.
	11. **Estonian participant (macro):** “If you give a task, you also have to allocate money. If the tasks are distributed among agencies, sufficient resources must be provided for that purpose. For example, it should be very clear who is responsible for what, we should find out if there is anything new in the scientific literature about distribution of tasks where there is a precise division between the agencies, so that there are no holes in the whole process”.
Prioritise prevention	12. **Bulgarian participant (meso):** “Every young girl that becomes sexually active until the women in their oldest age should have a mandatory check-up once a year, every year and a PAP smear every half year. The prophylactic PAP-smear should be free of charge, but also sanctions are needed if there is secured funding and the woman does not show up”.
Organised screening and Registry	13. **Bulgarian participant 1 (meso)**: “The idea that if there is such a national screening, it will be for everyone”.**Bulgarian participant 2 (meso):** “If there is a national screening, which is an overall one, there will be no such questions - which is not for me or where you took my name, address”.**Bulgarian participant 1 (meso):** “No, it’s in the beginning and then people get used to it and accept it”. (overlap of voices)**Bulgarian participant 2 (meso):** “If it is a state policy and health policy…”**Bulgarian participant 3 (meso):** “It must be a state policy”.
Adequate funding	14. **Romanian participant (meso):** “As we speak, I realize something I may not have fully grasped until now. We're discussing cervical cancer screening, but also considering funding for family doctors. The House can create this service with additional, though not substantial, payments, primarily for supporting staff. Without adequate funding, we risk missing the bigger picture. We're focusing on specific issues and losing sight of the overall goal.When we propose a prevention package, it should cover more than just cervical screening. It should include funding for positions like a secretary or medical registrar in family doctors’ offices. This would allow doctors to mobilize, inform and oversee, rather than be overburdened by administrative tasks.While a programme like this is necessary and useful, insufficient funding will strain family practices, eventually leading to burnout. Our discussions are valuable, but decision makers need to address the issue more holistically”.
Self-sampling—suspicion towards authorities	15. **Researcher**: “But a variant that has been pilot tested, would be for women to receive an envelope in the mail with a kit of self-testing, along with instructions, to take the sample, put it back in the envelope and send it to the laboratory or to be collected by those at XX centre. What would you think of such a situation?”**Romanian participants 1 (micro**): “The first question I ask myself: who asked me why in the Post Office?”**Romanian Participant 2 (micro**): “Exactly”.**Romanian Participant 3 (micro):** “How do you proceed?”**Romanian Participant 4 (micro):** “Yes, how is it done?”**Romanian Participant 3 (micro**): “Why did they put this to me?”**Romanian participant 5 (micro):** “I was really passing by one day; I was going up the stairs to the building I live, and there was a neighbour, very angry: who put this in my mail? At an age not even young, not very old…so no”.**Romanian participant 4 (micro**): “No, I don't know; for us this option does not work. The caravan works”.**Romanian participant 2 (micro**): “Or caravan, yes”.
	16. **Bulgarian participant (meso):** “I am not a specialist and maybe won’t be able to do what is required from me. I would rather see a specialist, in order to have it properly done.**Bulgarian participant (micro):** (I would) trust a health care specialist. I am not sure I am able to do it right, so that I can then send it forward”.
Self-sampling—convenient and empowering	17. **Estonian participant 1 (micro):** “Yes! First, she will do it at home, she does not have time for a doctor, she does not have to sit in lines, nothing. Secondly, it is neat, and then she will get the result. There are indeed many women who are complexed by the gynaecologist. Well, there it is, no matter how you look at it, and even I, when I sit, oh, I wish it would end soon… And you have to go. They can be provided by the family doctor or by the pharmacy. You can take it from there, and then you can send it wherever y want. There is data on where to send it. I took it and went”. **Estonian participant 2 (micro):** “It seems to me that this is a very good idea, I even lit up. You don't have to go anywhere. Made it, sent it off, and I'm comfortable. I mean, I am my own director, as they say”.

### Cluster 2: suggestions for improvement from participants in Denmark, Estonia, France, Italy, Portugal and Romania

In Denmark, Estonia, France, Italy, Portugal and Romania, the participants highlighted the need to tailor healthcare in general and CCS to the needs of vulnerable women and to increase collaboration and integration of services.

The participants deliberated the necessity of customising healthcare services to meet the unique needs of vulnerable populations while incorporating foundational values and concepts. This entailed adopting a holistic approach to vulnerable populations, respecting the individual’s autonomy and right to make choices, and providing individualised care (table 2, excerpts 7, 8, 9).

The participants suggested collaboration between and integration of social and health authorities and services, including patient data, to improve the CCS provision for vulnerable women. Greater interaction and collaboration between health and social services were debated in Denmark, Estonia and Portugal. In Italy, the integration of health mediators in the screening programme was highlighted (table 2, excerpt 10). In Estonia, the need for collaboration and a clear division of responsibility was emphasised to prevent gaps between services (table 2, excerpt 11). In Portugal and Romania, a system for integrating patient data from different services was highlighted.

### Cluster 3: suggestions for improvement from participants in Bulgaria and Romania

In Bulgaria and Romania, suggestions for improvement included prioritising and cultivating a culture of prevention, improvements in the provision of population-based and properly financed CCS programmes actively inviting all women for free screening.

The emphasis from participants was on prioritising and cultivating a preventive culture. In Bulgaria, the discourse involved suggestions to precisely define and comprehend the concepts of screening, and to implement a comprehensive anticancer plan along with annual prophylactic check-ups (table 2, excerpt 12).

In Bulgaria, the need for an organised screening programme was stressed including a regulatory framework and a screening registry (table 2, excerpt 13). In Romania, the implementation of a screening registry was similarly debated to monitor uptake, number of positive tests and colposcopies.

The participants issued the importance of a fully financed CCS service. Specifically, the Romanian discussions revealed a call for a more holistic approach to funding preventive healthcare in general to have all expenses covered including human resources (table 2, excerpt 14). Participants in all three countries stressed that women should have free access and health insurance covered.

### Perspectives on HPV self-sampling kits

In all countries except Bulgaria and Romania, the participants expressed positive reactions towards the idea of HPV self-sampling in CCS. The method was perceived as convenient, empowering, to possibly reduce fear and increase outreach among vulnerable women (table 2, excerpt 15).

In all countries, the participants also discussed the negative consequences of self-sampling. This included resistance to use the method due to fear of incorrect use, lack of trust in the validity of the test, loss of possibility for the medical doctor to make other observations during the test and preference for a specialist to perform the test. Bulgarian participants discussed that both healthcare provider and user had more confidence in a healthcare specialist to perform the test or explain how the test kit works (table 2. excerpt 16). Also, the participants argued that the method would not work for women with mental health issues, women who have suffered violence or abuse, women with low literacy or for women lacking trust in the authorities. Romanian participants specifically highlighted that receiving a self-sampling kit in the mailbox from authorities could arouse suspicion (table 2, excerpt 17).

## Discussion

This study identified stakeholders’ suggestions for the improvement of CCS for vulnerable women and mapped them according to similarities in clusters of countries.

Regardless of the level of implementation or lack of a population-based CCS programme, participants in all countries suggested increasing proximity and outreach work.

Proximity work aims to bring healthcare services closer to communities, reducing geographical barriers and emphasising direct engagement with vulnerable populations to understand their needs.^9^ The implications of providing direct access to CCS within the community might increase opportunistic screening and counterwork the increase in equality provided by population-based programmes. Mobile clinics and pop-up screening events that overcome transportation and logistical barriers are examples of proximity work.^8^

As suggested in the CUBs, outreach work may serve a critical role in CCS for vulnerable populations by promoting awareness and participation. Direct engagement with communities helps raise awareness about screening, dispel misconceptions, and address cultural barriers through community events, health fairs or educational workshops. Culturally competent care, including language interpretation services and diverse healthcare providers, fosters trust and confidence in the healthcare system. Through these approaches, outreach work may effectively promote CCS and improve health outcomes for vulnerable populations, as it has been shown in related areas.^19^,^22^

In the CUBs, we found contradictory reactions towards the use of self-sampling including fear of incorrect use of the test as well as the perspective that self-sampling could be empowering. The variation indicates the need for context-based increasing awareness and education which supports the idea of proximity and outreaching activities.

Previous studies have shown that emancipatory, empowering and participatory approaches are crucial when targeting vulnerable populations to improve healthcare. The American Deep South Network study proved effective awareness raising, education and use of cancer screening services among vulnerable populations through the development of a community of volunteer health advisors as research partners and by building partnerships at a community and state level.^23^ In a French study, an outreach community-based participatory intervention showed increased access to health insurance for unregistered immigrants.^24^ The need for outreach and proximity activities is further supported by Fuzzell and colleagues^25^ who found that mobile screening and employment of community workers can reduce barriers in CCS for vulnerable women. Outreach and proximity activities require capacity building at both provider and community levels. We found that suggestions of capacity building through training and education were connected to outreach activities to provide awareness and to increase skills to perform the test in local healthcare, social services and NGO settings. For example, So and colleagues^26^ explored the impact of training community health workers to provide awareness about breast and CCS and found increased awareness of cancer screening in minority groups. A scoping review aiming to identify the involvement of community health workers in the context of CCS in low- and middle-income countries showed that community-based programmes are feasible for raising awareness, knowledge and attendance in CCS. In our study, proximity and outreaching programmes, including opportunistic offering of the test, were considered by stakeholders as viable solutions with potential impact also in high-income countries as an addition to population-based programmes. To fit specific needs related to different vulnerable subgroups, the outreaching and proximity work needs to be inclusive, culturally sensitive and preferably based on participatory principles.

### Strengths and limitations

A main strength of the study is the multivoiced and Pan-European perspective provided through the involvement of stakeholders from end users to policy-makers.

Furthermore, the comparative design provides new knowledge of perspectives on resolutions across countries and vulnerable groups and shows the similar emphasis on outreach and proximity activities.

The synergy of the cross-disciplinary and national collaboration between research teams was a further strength of the study contributing cultural nuances and knowledge to the findings.

A limitation to the study was that data were translated into English by local research team members risking misinterpretation of meaning. However, the interpretations made by the first author were validated by the local research teams to reduce this risk.

We found that comparisons between stakeholder-level perspectives and vulnerable populations were impeded by the differences in the way the CUBS were organised and the vulnerable groups represented in each country. Therefore, the results represent a generalisation of perspectives across countries and vulnerable populations.

## Conclusion

Across European countries, vulnerable women are underserved in CCS causing preventable deaths. This study provides the views of stakeholders from seven European countries, with different professional backgrounds and social status on how CCS services can be improved.

In all countries, increased outreach activities and proximity work were emphasised as strategies for increasing awareness and education and establishing trustful relations.

In Denmark, Estonia, France, Italy, Portugal and Romania, participants focused on the need for a person-centred approach and tailored interventions to meet vulnerable women’s needs.

In Bulgaria and Romania, participants emphasised fully funded CCS pathways and access to women through screening registries as basic needs for improvement. The similarities and differences identified between countries are relevant to consider for policy and decision makers in the development of tailored programmes and for practitioners to be aware of their role in the encounters with vulnerable women. Based on this study, we recommend policy-makers to address the need for easy access and free screening and follow-up for all women. Furthermore, we recommend emphasising the development of outreaching-tailored programmes based on participatory insights into vulnerable women’s specific needs. Future research is needed to develop such programmes to increase vulnerable women’s participation in CCS across European countries.

## supplementary material

10.1136/bmjopen-2024-090631 online supplemental file 1

## Data Availability

Data are available upon reasonable request.
