# Supplementary material for: How can cervical screening meet the needs of vulnerable women? A qualitative comparative study with stakeholder perspectives from seven European countries
Source: BMJ Open. 2025 Jan 25;15(1):e090631. doi: 10.1136/bmjopen-2024-090631 (PMC11784119; doi:10.1136/bmjopen-2024-090631)
Supplement: online supplemental file 1 [file bmjopen-15-1-s001.docx]

Table 2. Suggestions for improvement elicited by stakeholders in each country.

| **Solutions** | **Bulgaria** | **Denmark** | | **Estonia** | **France** | **Italy** | | **Portugal** | **Romania** |
| --- | --- | --- | --- | --- | --- | --- | --- | --- | --- |
| **Provider level** | | | | | | | | | |
| **Theme: Education and training** | | | | | | | | | |
| **Education about vulnerability and communication skills** |  |  | | Include teaching about vulnerability in curriculum for medical students | Education for healthcare professionals about vulnerability, cultural awareness, tools to decrease prejudice. | Education for healthcare professionals about vulnerability and specific needs, improve communication skills, tools to avoid stigma. | | Education for healthcare professionals about vulnerability and specific needs, cultural awareness, tools to improve communication skills. |  |
| **Training to maintain skills and inform about CCS** | Train health mediators to inform. | Train nurses to perform the test.  Train health professional to offer the test when women are attending other medical services. | | Train general practitioners and social workers to inform about screening.  Train nurses and midwifes to perform the test and provide results. | Train nurses, midwifes and medical students to perform test.  Train midwifes to do colposcopies. | Cultural mediators, community workers and health workers, staff from associations.  Train general practitioners to inform about screening.  Train professional caregivers for women with disabilities and mental health issues to inform. | | Include other professionals to perform the test.  Train general practitioners to inform about screening. | Train health mediators in the community to inform and help invite vulnerable women.  Train social and medical mediators to inform about screening.  Follow-up training of health professionals performing the test. |
| **Theme: Tasks and the role of family and community** | | | | | | | | | |
| **Outreaching and proximity work** | Health mediators to inform by door-to-door outreach.  Use health mediators' knowledge of women's habits.  Provide psychological support to reduce fear and misconceptions.  Establish relationship to inform.  Healthcare staff to provide test-results. | Health professionals present at shelters.  Outreaching social  nurses. Provide access through already established relations.  Support from well-known providers to read invitations.  Establish trusting relations. | | Access to women through nurse practitioners.  Providers in established relations to take contact/invite.  Disabled people – through caregivers.  Support to read invitation.  A support person to help understand the test-result.  Information in the e-health portal needs to be commented by health professionals | Outreaching in the community to replace or as an addition to invitation letter. Outreaching mediators and other professionals.  Door to door contact.  Accompany women to test.  Support that fits vulnerable women's needs.  Test-results delivered face to face by a health professional or caregiver. | Cultural mediators to provide awareness in migrant reception centres.  Using existing outreach teams working on sex workers to establish relationships.  Accompany women to appointments | | Mediators within the community  Proximity work  Community relations  Peer educators in the community  Mediator should come with the nurse.  Support from a well know provider.  Support from a technician throughout the CCS pathway | Health mediators providing women with invitations in the community.  Employ support staff in primary care and community nurses and health mediators to help women fill in forms before the test. |
| **Family and community** | Family – the mother-in-law- plays a significant role. |  | | Family and community are important enablers. |  | Involvement of community leaders and influential figures in disseminating information. | |  | Awareness through family and friends, community, and priest. |
| **System level** | | | | | | | | | |
| **Theme: Underpinning values and approaches in healthcare** | | | | | | | | | |
| **An integration of vulnerable women's needs** |  | Focus on vulnerable women's needs.  Prioritise tailored practices – provide resources.  Expand healthcare services for vulnerable people to depend on more than zealots. | | Focus on vulnerable women's needs.  Individualised healthcare | Focus on vulnerable women's needs.  Empowerment | Focus on vulnerable women's needs.  Women have a right over their own body – empowerment.  Informed choice/Shared decision making.  A holistic approach to healthcare services for vulnerable women. | | Focus on vulnerable women's needs.  Tailor solutions and pathways to the needs of each group.  A general health project.  Informed choice – the right to choose.  Individualised healthcare. | Tailor interventions based on the demography in the area. |
| **Prioritise prevention** | Prioritise prevention.  Defining the concepts of prevention and screening clearly  A national anti-cancer policy  A yearly prophylactic check-up for all women |  | | Promote a culture of prevention. |  |  | |  | Decisionmakers should look at prevention more holistically |
| **Theme: Organisation and administration of CCS** | | | | | | | | | |
| **Organised screening and Registry** | An organised screening programme.  A regulatory framework  A screening registry |  | |  |  |  | |  | A screening registry  A central invitation system |
| **Collaboration and integration of services** |  | Supporting administrative tools for health professionals and social workers | | Communication between authorities  Clear division of responsibility | An integrated administrative CCS pathway | An integration of the screening programme with cultural mediation | | Communication between associations supporting vulnerable women.  Integration of data from public and private screening | A system that integrates patient data |
| **Funding** | Centrally financing of screening  Cover the health insurance for the most vulnerable groups.  Free access for all women |  | |  | Fully financed CCS pathway |  | |  | A holistic approach to funding  Sufficient funding for each part of the CCS pathway from awareness to follow up.  Free access for all women |
| **Theme: Awareness** | | | | | | | | | |
| **Oral/Written/Other** | Oral:  door to door, TV adds. social media  Written:  email, posters, leaflets. | Oral:  at schools, youth clubs  public events, shelters, substance abuse clinics. social media, videos, animated films  Written:  Leaflets, billboards, adds at busses/busstops  Other:  national campaigns | | Oral:  at schools, social events,  peer learning through experiental stories, TV adds, social media.  Written:  websites, links at e-health portal, leaflets, regional and local adds at buses/busstops  Other:  make screening fashionable, fancy badges, nudging behaviour, national screening day | Oral:  at schools and communities through social services, healthcare vans, TV adds, social media,  voice messages,  influencers  Written:  Text messages  Other:  shared decision-making tools. | Oral:  peer learning – sharing experiences. | | Oral:  group sessions for women., TV adds,  oral information at interactive platform  Influencers | Oral:  provided from caravans, social media. |
| **Requirements and content** | Clear and coherent information | Provide an overview of the CCS pathway | | Clear and coherent information Co-constructed and adjusted information and invitations  Provided in different languages.  Use of non-frightening words and pictograms  Easily accessible information at webpages and e-health  Provide an overview of the CCS pathway.  That screening is for free, and treatment is effective | Provided in different languages Co-constructed invitations. | Provided in different languages.  Culturally mediated information  information about cancer prevention and all types of screening at once | | Use of simple language and figures The consequences of the disease.  Breaking stigma | That screening is for free, and treatment is effective. |
| **Accessibility** | | | | | | | | | |
| **Invitations** |  | Prescheduled appointment  Personal reminders  A video invitation | | Prescheduled appointment  Personal reminders  Personal invitations  A no show fee | Social media messaging channels | Invitation with QR code providing further information. |  | | Prescheduled appointment  A reminder system at the family doctor  Social media messaging channels.  Personal invitation coming from the family doctor.  Invitations sent to mailbox. |
| **Easy access** | Mobile unit | Mobile units  Drop-in centres  Access to screening at substance abuse clinic, shelter, and other medical services  An all-round healthcare check-up including CCS at substance abuse clinics. | | Mobile units  Parking possibilities  When women are attending other healthcare services | Mobile units with midwifes.  Local test facilities  Drop-in screening without registration.  When women are attending other healthcare services. | Street units  Local facilities  Accommodate schedules to user's needs | Mobile units  Local facilities  Proximity work  Less bureaucracy  Reinforce availability.  Self -registration. | | Mobile units  Local access  Convenient access to facilities  Less bureaucracy |
| **Access to female doctor** |  |  | |  |  | Make it possible to choose a female doctor |  | | Make it possible to choose a female doctor |
| **Access to test- results** | Text message, email, or postal mail |  | | Negative and positive results should be communicated to women.  Test results should be written in an easy and comprehensible manner |  |  |  | |  |
| **Self-sampling** | | | | | | | | | |
| **Positive reactions** |  | Convenient for women.  Less anxiety provoking. | | Convenient for all women and in particular young women, women with disabilities, special needs, or women who have experienced sexual abuse.  Might be perceived as personalised screening when the test is sent to home address. | Convenient for women. | A good option in addiction services.  A tool for empowerment and awareness raising. | Should be available for all women.  It solves problems related to shame and embarrassment.  Mothers could buy for daughters.  A way of saving lives | |  |
| **Negative reactions** | Resistance to use test.  Fear of incorrect use.  Lack of trust in validity of test  Loss of other observations during test.  Preferences for a specialist. | Resistance to use test. | Fear of incorrect use. | | It will not be a priority for women living with vulnerable situations.  Loss of contact with the women. | Fear of incorrect use.  Not appropriate for women with mental health issues, victims of violence  Logistic challenges with sending samples through postal services.  Cannot replace sampling in clinics. | Fear of incorrect use.  Loss of other gynaecological observations.  Women with low literacy will have less tendency to accept the method.  A challenge for women with Muslim beliefs and perspectives on their bodies.  Professionals losing contact with women. | | Lack of trust in validity of test.  Lack of trust in test kit when sent via postal services |
