# Supplementary material for: How can cervical screening meet the needs of vulnerable women? A qualitative comparative study with stakeholder perspectives from seven European countries
Source: BMJ Open. 2025 Jan 25;15(1):e090631. doi: 10.1136/bmjopen-2024-090631 (PMC11784119; doi:10.1136/bmjopen-2024-090631)
Supplement: Supplementary file 2 [file bmjopen-2024-090631.reviewer_comments.pdf]

PEER REVIEW HISTORY

BMJ Open publishes all reviews undertaken for accepted manuscripts. Reviewers are asked to complete a checklist review form and are provided with free text boxes to elaborate on their assessment. These free text comments are reproduced below.

ARTICLE DETAILS

Title (Provisional)

How can cervical screening meet the needs of vulnerable women? A qualitative comparative study with stakeholder perspectives from seven European countries.

Authors

Bøje, Rikke Buus; Bardou, Marc; Mensah, Keitly; Rico Berrocal, Raquel; Bonvicini, Laura; Iorio, Giusy; Auzzi, Noemi; Taut, Diana; Pașca, Nicoleta-Monica; Tisler, Anna; Reintamm, Kerli; Uusküla, Anneli; Teixeira, Margarida; Firmino-Machado, João; Amorim, Mariana; Baia, Ines; Lunet, Nuno; Panayotova, Yulia; Kotzeva, Tatyana; Todorova, Irina; Andersen, Berit; Kirkegaard, Pia; CBIG-SCREEN consortium, CBIG-SCREEN consortium

---

VERSION 1 - REVIEW

**Reviewer**	**1**
**Name**	**AlAbdulKader, Assim M.**
**Affiliation**	**Imam Abdulrahman Bin Faisal University**
**Date**	**28-Jul-2024**
**COI**	**None**

Overall, the manuscript is well-written and thoroughly researched and provides valuable insights into improving cervical cancer screening for vulnerable women. Addressing the recommendations below will strengthen the article and enhance its impact on the academic and medical communities.

Abstract: It outlines the paper sections and provides a comprehensive summary of the study, emphasizing the qualitative comparative approach and the involvement of stakeholders across seven European countries. Recommendations: To clarify the findings, add specific data points or statistics to the abstract.

Introduction: The introduction effectively sets the stage for the research, highlighting the importance of cervical cancer screening (CCS) and the disparities in screening attendance among vulnerable women in Europe. Recommendations: Provide a brief overview of the CBIG-SCREEN project earlier in the introduction to give readers immediate context about the study's framework.

Methods: The methodology section is detailed and robust, describing the qualitative participatory approach and using Collaborative User Boards (CUBs) for data collection. Including diverse stakeholders at micro, meso, and macro levels is a strength, ensuring a comprehensive perspective. Recommendations: Clarify the criteria for selecting the seven countries and elaborate on any potential biases the selection process introduces.

Results: The results are presented clearly, focusing on provider and system-level suggestions for improving CCS for vulnerable women. The engagement of 120 participants across seven countries provides a broad dataset. Recommendations: Include direct quotes from participants to add depth and personal perspectives to the findings. Ensure that all tables are referenced in the text for more straightforward navigation.

Discussion: The data will support the emphasis on building trust, tailoring healthcare services, and ensuring free access to screening. The limitations section appropriately addresses the cross-country variation in participant numbers and meeting structures. Recommendations: Expand on the implications of the findings for future research and policy development. Discuss any unexpected findings and their potential impact on the study's conclusions.

Conclusion: The study reinforces the need for targeted interventions and policy changes to improve CCS access and participation among vulnerable women in Europe. Recommendations: Based on the study's findings, highlight specific action points or recommendations for stakeholders and policymakers.

**Reviewer**	**2**
**Name**	**Mulcahy Symmons, Sophie**
**Affiliation**	**University College Dublin, School of Nursing, Midwifery & Health Systems**
**Date**	**12-Aug-2024**
**COI**	**NA**

A very interesting read with useful application for researchers working in the field of cervical cancer screening promotion and health equity which is complimentary to your recently published paper on the barriers to cervical screening among vulnerable populations. My comments are suggestions for clarification.

Introduction

Aim is clear but could give more context that this is the second of two aims of the study, the first of which is published and should be cited here.

What was the rationale for splitting this study into two papers?

Methods

The methods are written very similarly to your recently published study (Bøje et al., 2024), better referencing is needed to highlight that this was the same study and method but with different objectives. It may be worth explaining briefly in the Introduction/aims section to frame the methods. I see the reasoning for explaining the methods in detail again here, but you could reference the first paper and just detail the key information and what was different about this analysis.

It is not clear to me what a Collaborative User Board is. I think it would benefit from more explanation and/or a supplementary file with details of a meeting agenda or topic guide for the focus groups and what techniques you used for a “co-creative process”. This would aid repetition of this study.

I would also be interested to know how previous sessions on the barriers of cervical screening informed subsequent meetings on solutions.

How do you define “vulnerable”?Is it those that are less likely to participate in screening or a broader social determinants of health definition? When talking about vulnerable groups, you could reference Table 2 with the list of what populations are included as “vulnerable” and the numbers of participants in each population group.

Was there any reason for using Bulgaria, Denmark and Estonia to develop your framework or was it a random subset of countries?

It is not clear to me how Public Patient Involvement was part of this study. It seems people with lived experience were just participants in the study and had no role in development of the study itself. This should be clarified whether those with lived experience had a greater role to play in the research beyond participation in the CUBs.

Results

It would be useful if your framework analysis also looked at similarities/differences between macro-, meso-, and micro-level participants. Did micro-level participants have different things to say about what would support them to attend screening? Were there any differences across the vulnerable populations? Additional analysis of the dataset could be considered in the future to elucidate this.

Discussion

There was no mention that vulnerable populations have unique needs and can’t always be reached in the same way. The discussion would benefit from explaining the limitations of this research by not comparing by population groups and therefore generalise the needs of vulnerable populations. Suggested supports identified in the results would need to be explored for acceptability and tailored to each population. Does the literature show that one type of intervention work particularly well over another intervention or compared to another vulnerable population?

The strengths and limitations section could describe the limitation of not using the framework analysis to look at similarities/differences between macro, meso, and micro level participants or by vulnerable populations as it generalises the needs of vulnerable populations and may make it difficult to interpret your results for future work to develop tailored interventions that are acceptable to each vulnerable population.

VERSION 1 - AUTHOR RESPONSE

Reviewer	Comment	Response
#1 Dr. Assim M. AlAbdulKader, Imam Abdulrahman Bin Faisal University	Abstract: To clarify the findings, add specific data points or statistics to the abstract.	We are unable to provide statistic data owing to the qualitative design. We are not sure what is meant by specific data points?
	Background: Provide a brief overview of the CBIG-SCREEN project earlier in the introduction to give readers immediate context about the study's framework.	We have moved the information about CBIG-SCREEN earlier in the introduction (Line 117-122)
	Methods: Clarify the criteria for selecting the seven countries and elaborate on any potential biases the selection process introduces.	We have clarified the inclusion criteria (Line 166-169)
	Results: Include direct quotes from participants to add depth and personal perspectives to the findings. Ensure that all tables are referenced in the text for more straightforward navigation.	We have provided quotes corresponding to the results in Table 3. We decided to maintain the quotes in the table to make a more fluent presentation of the results.
	Discussion: Expand on the implications of the findings for future research and policy development. Discuss	We have addressed this issue in the

	any unexpected findings and their potential impact on the study's conclusions.	conclusion (Line 422-425)
	Conclusion: Based on the study's findings, highlight specific action points or recommendations for stakeholders and policymakers.	See above
Reviewer # 2 Ms. Sophie Mulcahy Symmons, University College Dublin		
	Aim is clear but could give more context that this is the second of two aims of the study, the first of which is published and should be cited here.	We have clarified this information (Line 121-122 and 157-158)
	What was the rationale for splitting this study into two papers?	Owing to a large dataset and the risk of losing the qualitative nuances, we divided the study into two aims and two papers.
	The methods are written very similarly to your recently published study (Bøje et al., 2024), better referencing is needed to highlight that this was the same study and method but with different objectives. It may be worth explaining briefly in the Introduction/aims section to frame the methods. I see the reasoning for explaining the methods in detail again here, but you could reference the first paper and just detail the key information and what was different about this analysis	We have made a comprehensive re- organisation of the method section, now referring to the previous study for a more detailed description. **See method section**
	It is not clear to me what a Collaborative User Board is. I think it would benefit from more explanation and/or a supplementary file with details of a meeting agenda or topic guide for the focus groups and what techniques you used for a “co-creative process”. This would aid repetition of this study.	We have added extra information about the intention with CUBS (Line 159-161). Furthermore, we are working on a methodological paper to

		support repetition.
	I would also be interested to know how previous sessions on the barriers of cervical screening informed subsequent meetings on solutions.	More information has been added (Line 184-187).
	How do you define “vulnerable”?Is it those that are less likely to participate in screening or a broader social determinants of health definition? When talking about vulnerable groups, you could reference Table 2 with the list of what populations are included as “vulnerable”	Information has been added (Line 177-180)
	Was there any reason for using Bulgaria, Denmark and Estonia to develop your framework or was it a random subset of countries?	We have reorganised the method section and this part has been deleted.<br>The selection was random based on alphabetical order.
	It is not clear to me how Public Patient Involvement was part of this study. It seems people with lived experience were just participants in the study and had no role in development of the study itself. This should be clarified whether those with lived experience had a greater role to play in the research beyond participation in the CUBs.	We have deleted the sentence in the methods section and elaborated on the position in the mandatory PPI section of the paper.
	It would be useful if your framework analysis also looked at similarities/differences between macro-, meso-, and micro-level participants. Did micro-level participants have different things to say about what would support them to attend screening? Were there any differences across the vulnerable populations? Additional analysis of the dataset could be considered in the future to elucidate this.	This is a good idea. We are planning on a future publication based on national data only, where the differences between stakeholder perspectives will be included.
	There was no mention that vulnerable populations have unique needs and can’t always be reached in the same way. The discussion would benefit from explaining the limitations of this research by not	Thank you for highlighting this. We have addressed this

	comparing by population groups and therefore generalise the needs of vulnerable populations. Suggested supports identified in the results would need to be explored for acceptability and tailored to each population. Does the literature show that one type of intervention work particularly well over another intervention or compared to another vulnerable population?	in the discussion and strength and limitation section (391-393 and 406 - 409).
	The strengths and limitations section could describe the limitation of not using the framework analysis to look at similarities/differences between macro, meso, and micro level participants or by vulnerable populations as it generalises the needs of vulnerable populations and may make it difficult to interpret your results for future work to develop tailored interventions that are acceptable to each vulnerable population.	See above

VERSION 2 - REVIEW

Reviewer 2

Name Mulcahy Symmons, Sophie

Affiliation University College Dublin, School of Nursing, Midwifery & Health Systems

Date 10-Dec-2024

COI

Well done revising this manuscript.

I have just two suggestions before publication:

1. I would recommend you include brief information on ethics approval and consent that had been removed in the Methods section as it can be overlooked in the Ethics statement at the end of the manuscript.

2. There is some repetition in the results, particularly in relation to HPV self-sampling, and I suggest you consider what needs to be summarised on pgs 8-9 and what should be described in more details pgs 10-12.
